# Precise delivery of medical gases by engineered nanomedicine for enhanced liver fibrosis therapy

**DOI:** 10.1016/j.mtbio.2026.103104

**Published:** 2026-04-09

**Authors:** Peiyuan Tian, Jinping Wang, Haohan Yang, Zhenpu Jiang, Jinshuai Xu, Xueli Xu, Nengyi Ni, Teng Liu, Xiao Sun

**Affiliations:** a Nanchang University Queen Mary School, Nanchang, 330006, China; b School of Biological Science and Technology, University of Jinan, Jinan, 250022, China; c Shandong Cancer Hospital and Institute, Shandong First Medical University & Shandong Academy of Medical Sciences, Jinan, 250117, China; d School of Science, Shandong Jianzhu University, Jinan, 250101, China

**Keywords:** Liver fibrosis, Medical gas, Enhanced therapeutics, Drug delivery

## Abstract

Liver fibrosis, a significant global health issue, results from sustained liver injury and is characterized by progressive damage to the liver. Treating liver fibrosis poses considerable challenges due to difficulty in reversal and frequent association with complex, chronic liver injuries. Recently, medical gas therapies have emerged as a promising novel approach for addressing liver fibrosis. The primary gases involved include carbon monoxide (CO), hydrogen (H_2_), nitric oxide (NO), hydrogen sulfide (H_2_S), sulfur dioxide (SO_2_), and oxygen (O_2_). Several gases exist as endogenous gasotransmitters, thereby inherently capable of participating in known cellular signaling pathways that can be exploited for therapeutic purposes. The principal therapeutic advantages of these gases revolve around the attenuation of key features of liver fibrosis. They exert antioxidant and anti-inflammatory effects, inhibit hepatic stellate cell (HSC) activation and cytokine production, neutralize reactive oxygen species (ROS), and prevent hepatocyte apoptosis. Leveraging on the unique, attractive properties of medical gases, this review examines current scientific findings and investigates the use of advanced delivery modalities, including molecular donors and nanotechnology-based systems, to enhance the specificity of gas therapy for liver fibrosis.

## Highlights

• Liver fibrosis therapies urgently need innovative strategies and novel approach.
• Medical gases possess inherent ability to exert multi-pronged therapeutic effects.
• Advanced delivery systems and carriers are needed to control dosage and targeting.
• This review discusses current state of medical gases for treating liver fibrosis.

## Graphical abstract

**Figure:**
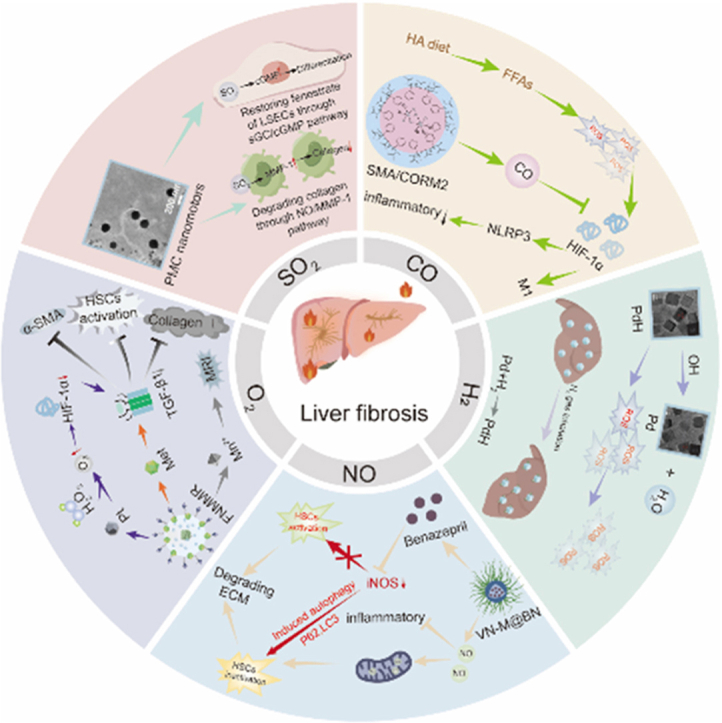

## 1 Introduction

### 1.1 Global burden of liver fibrosis

**Fig. 1:**
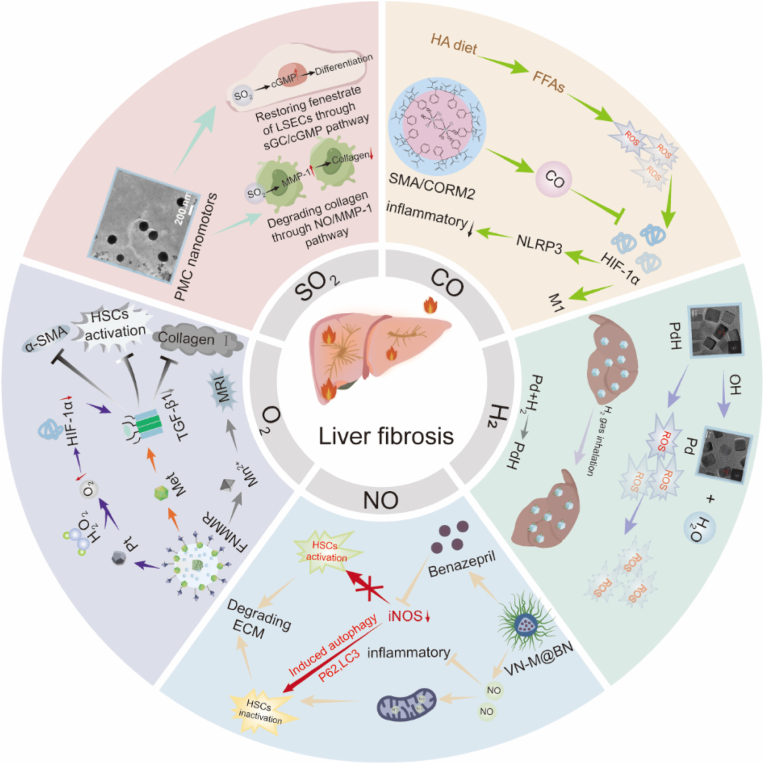
Graphical illustration representing the application of medical gas therapy in liver fibrosis.

Liver fibrosis, which results from chronic liver disease, is dynamic and potentially reversible before further progression. It can be caused by chronic viral hepatitis (HBV and HCV), alcoholic liver disease (ALD), metabolic dysfunction-associated steatotic liver disease (MASLD), autoimmune hepatitis, or cholestatic injury [1,2]. Chronic and persistent inflammatory injury caused by activated hepatic stellate cells (HSCs) are critical during liver fibrosis progression [3,4]. These activated cells lose their quiescent state and turn into contractile myofibroblast-like cells that increase in number and produce extracellular matrix (ECM) proteins [5]. Activated macrophages and injured hepatocytes also send signals that promote ECM deposition and fibrosis [6,7]. Excess ECM deposition will then lead to fibrous tissue proliferation that distorts liver architecture, alters blood flow, and reduces metabolic function [8]. Furthermore, cross-linked collagen traps tissue and provides a scaffold for further HSC activation [9]. Uncontrolled inflammation and fibrosis can eventually progress to cirrhosis, with heightened risks of progression to liver failure, hepatocellular carcinoma, and portal hypertensive vascular complications. The progression to advanced fibrosis and cirrhosis also results in significant vascular changes, formation of regeneration nodules, and portal hypertension [10,11]. Current therapies regarding liver fibrosis focus on disrupting the cycle of HSC activation and ECM deposition [12] and primarily slow disease progression, highlighting the need for new therapies that target the core mechanisms of fibrogenesis [13]. While addressing the underlying causes, such as viral hepatitis, can partially reverse or slow fibrosis, few pharmacological options currently exist to inhibit the fibrotic process [14,15]. Consequently, research into new therapies for liver fibrosis focuses on intercepting the mechanisms behind scar tissue formation (see Fig. 1).

### 1.2 Current therapeutic interventions for liver fibrosis

Presently, liver fibrosis identified in patients is addressed through an assortment of therapeutic interventions. For patients with HBV and HCV, etiological treatments first address the underlying causes of liver injury. HBV is treated with antiviral drugs such as Tenofovir [16], while HCV is treated with regimens such as a sofosbuvir/velpatasvir [17] antiviral combination. Antiviral treatments in patients are expected to be implemented for many years till undetectable viral loads, but result in a varying degree of liver fibrosis improvement, where some patients experience good fibrotic regression while others progress instead [18,19]. Meanwhile, in patients diagnosed with ALD, the primary intervention is ceasing alcohol consumption to improve liver function (where possible) and patient survival [20]. However, there are no current pharmacological solutions for liver fibrosis from alcoholic damage [21,22]. Liver fibrosis arising under MASLD (specifically nonalcoholic steatohepatitis, NASH) has an approved pharmacological intervention, in the form of the drug Resmetirom, which is an agonistic drug of thyroid hormone receptor-beta (THR-β) achieving liver fat reduction and fibrosis improvement [23]. Clinically established drugs such as the sodium-glucose cotransporter 2 (SGLT2) inhibitors [24] or glucagon-like peptide-1 (GLP-1) agonists [25] could possibly attain alternate usage in liver fibrosis but most drugs or regimens remain investigational, under clinical trials. In general, advancement of fibrosis will progress towards irreversible liver cirrhosis [14], where treatment options become seriously limited. Presently, approved anti-fibrotic pharmacological solutions remain rare. In one example, anti-fibrotic candidate Selonsertib failed at phase III investigation and could not alleviate fibrosis in real patients [26] despite very notable effects in vivo, possibly due to compensatory mechanisms in the complex fibrotic niche [27,28]. Thereby, broadening the arsenal of anti-fibrotic drugs that can alleviate this heterogeneous condition is essential, especially therapeutics with innovative approaches.

### 1.3 Emergence of medical gas therapy

Medical gas therapy represents a modern approach to targeted treatment for liver fibrosis, with promising results. Several studies in preclinical and experimental settings have confirmed the medical and therapeutic properties of gases [29,30]. Dynamic gases and their active compounds can protect cells, perform scavenging activities, reduce inflammation, and inhibit excess scar tissue formation in the liver [31]. These compounds take part in liver injury and repair mechanisms, along with many cellular signaling pathways. When delivered in controlled quantities, carbon monoxide (CO) offers protective benefits by modulating hypoxia-inducible factor-1α (HIF-1α), which also plays a role in fibrogenesis. CO-releasing micelles in animal studies targeting fibrotic, inflamed livers described reductions in inflammation and fibrosis, without systemic toxicity [32]. Hydrogen (H_2_) acts as a selective antioxidant, neutralizing potent generators of oxidative stress such as hydroxyl radicals and peroxynitrite. H_2_ applications in liver fibrosis are associated with decreased liver inflammation, improved metabolic control, and reduced reactive oxygen species (ROS) in activated HSCs. H_2_ gas is relatively safer than most common therapeutic agents, which is an important advantage [33,34]. Nitric oxide (NO) has a complex role in the liver; at higher levels, it stimulates inflammation, whereas, when delivered specifically, it can reduce fibrosis. HSC-targeted NO-loaded nanoparticles inhibit TGF-β signaling, decreasing fibrogenic gene expression. Light-induced NO release reduces liver inflammation and collagen synthesis, demonstrating the positive therapeutic potential of NO [35,36]. Hydrogen sulfide (H_2_S), as an endogenous gasotransmitter, has strong anti-fibrotic capabilities by inhibiting HSC activation through reduced expression of α-SMA and preventing the transition of quiescent cells into myofibroblasts. Defective H_2_S metabolism during liver injury presents therapeutic potential with external H_2_S donors [37]. Besides its benefits for therapy, sulfur dioxide (SO_2_) remains categorized as a pollutant, along with cytokines, ECM, and collagen in fibrotic liver tissue [38]. However, the use of advanced carrier designs may significantly enhance the safety and effectiveness of these types of gases. Gas exposure is minimized through nanotechnology-based carriers designed for controlled, targeted release [39,40]. The integration of nanomedicine with medical gas therapy marks a major step toward clinical application through improved stability and precision in targeting and dosing [41].

### 1.4 Role of nanotechnology in enhancing medical gas therapy

Nanotechnology supports the advancement of medical gas therapy, especially through endowing it with spatiotemporally controlled delivery. Medical gases often suffer from rapid diffusion and lack of targeting [42,43], along with certain gas-specific challenges such as unwanted toxic exposure of CO [44] or low solubility of H_2_ [45]. In the case of NO, its extremely short half-life (in milliseconds) and non-specific delivery first inspired NO donors that release NO under stimuli such as pH or glutathione (GSH) [46], or prodrugs [47] that release the gas under specific physiological condition such as enzyme exposure. Similar to other endogenous gases, NO exhibits multiple roles within the body. NO is highly reactive and can yield ROS or reactive nitrogen species that exert oxidative damage on normal cells, thereby rendering the development of NO delivery systems important [48]. Nanotechnology has further advanced these NO donor innovations by enabling their encapsulation, for example, within a PEGylated liposome [49]. In this form, the NO donor demonstrates improved accumulation in tumors for up to 48 h, achieving targeted delivery and biological concentration at the desired sites, instead of aimless transport and premature release at healthy tissues. Controlling release of NO donors is also achieved through various forms of stimuli response [[50], [51], [52]]. In the case of H_2_S, this gasotransmitter can be exogenously donated for therapeutic effects with species such as GYY4137 [53] or *N*-acetyl cysteine [54]. Nanomedical innovations help to improve targeting and delivery of H_2_S [55]. For example, the clinically available H_2_S donor anethole trithione was loaded into a magnetic nanoliposome, where external magnetic field and ultrasound stimuli both assisted in its delivery and release [56]. In another work, a polysulfide-based donor species was encapsulated into amphiphilic conjugated polymers, where the system could respond to glutathione levels in tumors, release H_2_S, and also underwent photothermal therapy (PTT) to maximize killing effects [57]. For CO, CO releasing molecules (CORMs) can be encapsulated into core shell nanostructures with light stimulated release and targeting ability such as to folic acid [58,59]. Nanosystems incorporating CORMs include structures such as micelles, dendrimers, metal-organic frameworks, and inorganic core shell structures [60]. Inappropriate release of high concentrations of CO will otherwise bind to hemoglobin in blood and reduce oxygen transport in the body, resulting in CO poisoning [61]. Precise nanomaterial formulations of CORMs further mitigate challenges of using CO by offering opportunities to facilitate controlled release or unique stimuli-response due to the delivery carrier design, biological enrichment (such as through passive targeting), and improved biocompatibility [62]. As with other gases, nanotechnology also allowed for combination therapies involving multiple medical gases, such as CO with NO release, for maximal synergistic impact upon the target sites [63]. Therefore, nanotechnology is crucial for overcoming key traditional limitations of medical gases—including their short half-lives, low solubility, and lack of targeting capability. It achieves this by offering novel systems that shield them from degradation, enable targeted delivery and unique surface modifications, controlled release in response to specific stimuli, and support combination therapies with other gases or drugs.

This review examines the treatment of liver fibrosis using medical gases. It emphasizes the molecular mechanisms underlying the anti-fibrotic effects of medical gases, including reducing oxidative stress, suppressing inflammatory cascades, preventing hepatocyte apoptosis, and directly inhibiting hepatic stellate cell activation. It also addresses the challenges of translating these findings into clinical practice, particularly the potential benefits of novel delivery methods, such as gas-releasing compounds and nanocarriers, to enhance targeting and dose precision. By examining current experimental and preclinical studies, this review aims to strengthen the scientific basis for medical gas therapy and discuss their potential clinical applications.

## 2 Engineered nanomedicine with precise delivery of medical gases for enhanced liver fibrosis therapeutics

Medical gas therapy has recently gained recognition as a transformative alternative to traditional pharmacological methods [29]. This approach employs endogenous gases such as CO, H_2_, NO, H_2_S, and SO_2_, which are essential signaling molecules that readily diffuse across biological membranes. The efficacy of these gases arises from their multimodal mechanisms, targeting multiple pathological pathways simultaneously through potent antioxidant, anti-inflammatory, anti-fibrotic, and anti-apoptotic effects [64,65]. Mechanistically, gasotransmitters influence vital cellular functions by regulating key signaling pathways; for instance, specific gases activate the Nrf2-mediated antioxidant response to reduce oxidative damage while also suppressing NF-κB-driven inflammatory processes [44,45]. A fundamental anti-fibrotic mechanism involves their direct inhibition of HSC activation and proliferation, preventing excessive extracellular matrix formation. The original endogenous roles of gases make them especially poised to address oxidative stress, chronic inflammation, and core drivers of fibrogenesis, making medical gas therapy a highly versatile for complex, multifactorial diseases such as liver fibrosis, providing a comprehensive therapeutic approach that surpasses the limitations of single-target pharmaceutical therapies [66,67].

### 2.1 Therapeutic potential of CO in liver fibrosis

**Fig. 2:**
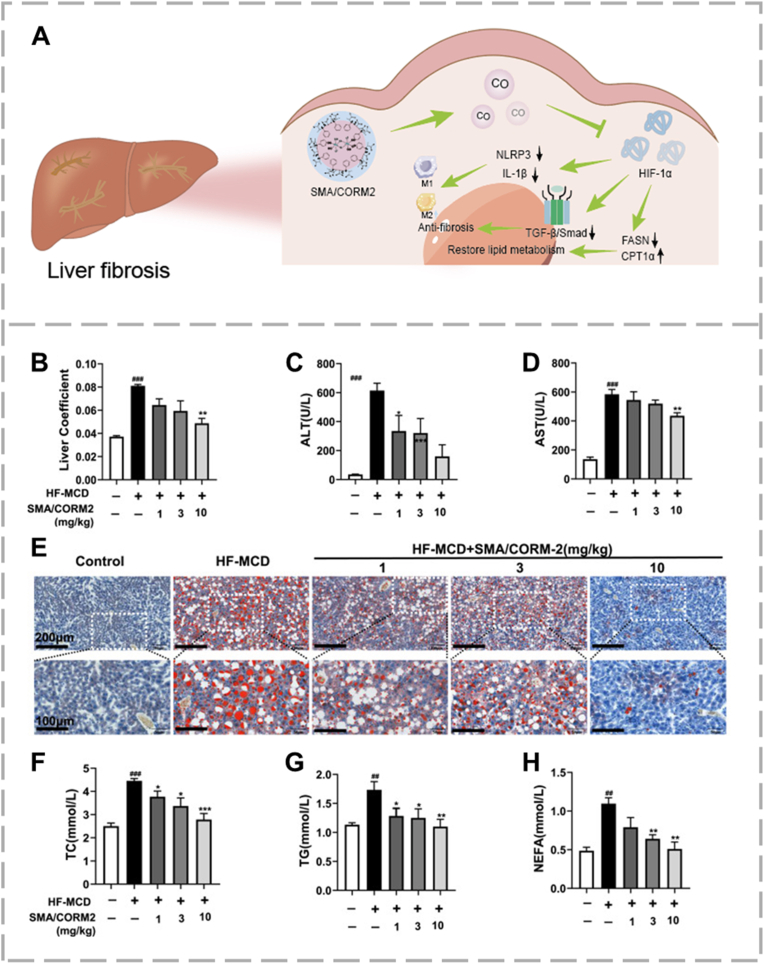
Evaluation of the therapeutic efficacy of SMA/CORM2 micelles in a MAFLD model. (A) Low-dose CO released shows therapeutic effect on liver fibrosis. (B) Liver-to-body weight ratio. Serum biomarkers of (C, D) hepatic injury (E) Photomicrographs of Oil Red O-stained liver sections revealing hepatic steatosis and (F-H) lipid metabolism, reproduced from Ref. [72] with permission. (For interpretation of the references to color in this figure legend, the reader is referred to the Web version of this article.)

CO is a colorless, odorless gas typically known for its toxicity because it binds to hemoglobin and interferes with oxygen transport [68]. However, low doses of exogenous CO show strong anti-fibrotic effects [69]. Metabolic dysfunction-associated fatty liver disease (MAFLD) covers a spectrum of conditions that can progress from steatohepatitis to cirrhosis and hepatocellular carcinoma. The HIF-1α signaling pathway has been identified as a critical upstream regulator in the progression of MAFLD [70,71]. In Fig. 2, Cui et al. designed for a polymeric micelle formulation of the CO-releasing molecule CORM2, encapsulated within styrene-maleic acid copolymer (SMA) [72]. The resulting SMA/CORM2 micelle is water-soluble and released CO slowly without requiring external activation. The nanocarrier design benefited CORM2 through extending its half-life by 35-fold and improved aqueous solubility (>50 mg/mL), providing passive accumulation at the liver via enhanced permeability and retention (EPR) effect, controlled sustained release (releasing CO across 48-72 h as opposed to mere minutes) and, protected CORM2 from premature degradation and toxicity in blood. In a MAFLD diet-based mouse model, SMA/CORM2 exhibited its therapeutic effects mainly through strong suppression of HIF-1α expression, significantly improving hepatic steatosis and slowing disease progression in MAFLD. CO has also been found to induce stress granule formation through activation of the integrated stress response [73]. A 2023 study showed that CO-dependent activation of tristetraprolin decreases levels of plasminogen activator inhibitor-1 within stress granules, helping to reduce age-related NAFLD [74]. Therefore, exploring how CO interacts with molecular pathways involved in liver fibrosis may lead to new treatment options for NAFLD/MAFLD.

### **2.2** **Therapeutic potential of H_2_ in liver fibrosis**

**Fig. 3:**
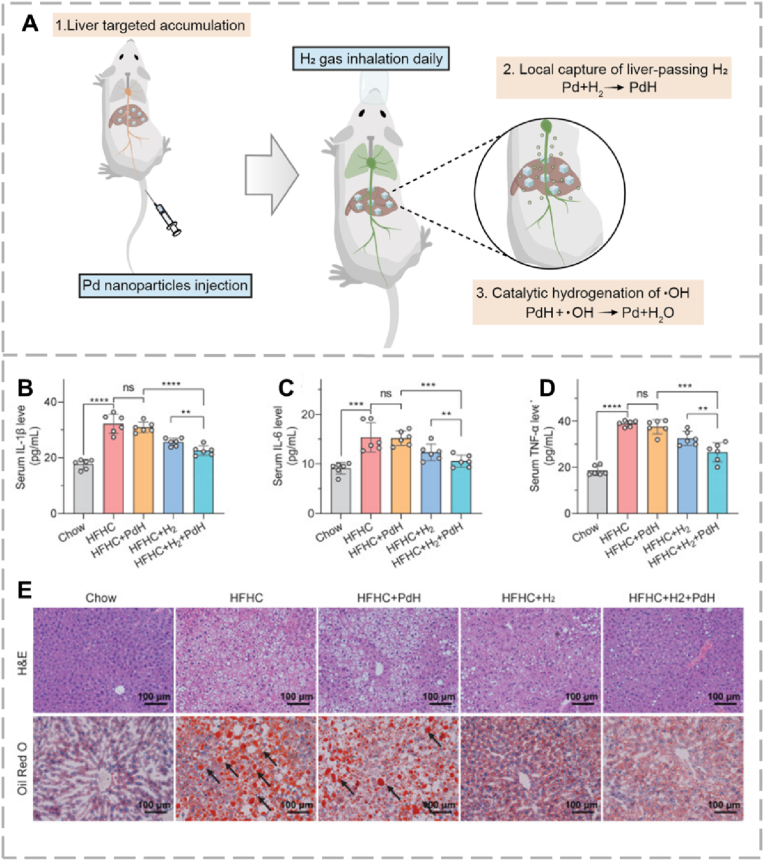
(A) Schematic diagram showing how fibrosis is induced through oxidative stress, ROS and pro-inflammatory cytokines, while H_2_ scavenges hydroxyl radicals (•OH) to restore liver function. (B–D) Serum IL-1β, IL-6, and TNF-α levels are shown. (E) Representative liver H&E and Oil Red O staining (scale bar = 100 μm). H_2_ and PdH + H_2_ treatments reduced steatosis and histopathological injury (arrows) induced by the HFHC diet, reproduced from Ref. [78] with permission. (For interpretation of the references to color in this figure legend, the reader is referred to the Web version of this article.)

Molecular H_2_ shows broad-spectrum anti-inflammatory and cytoprotective effects, mainly through its ability to selectively neutralize the cytotoxic hydroxyl radical [34] ). Its high biosafety profile and therapeutic potential have been confirmed in models of inflammation-related diseases, including hepatitis [75]. Several clinical trials suggested that consuming hydrogen-rich water may improve inflammation and metabolic dysfunction in patients experiencing chronic liver disease [76]. Inhalation of H_2_ is another common administration route being studied for treating chronic liver disease [77]. A major advantage of H_2_ is its possible superior safety compared to traditional medications for chronic liver disease. To improve its therapeutic efficacy, targeted delivery methods are being developed. One such approach*,* introduced by Tao et al., used palladium/palladium hydride (PdH)-based nanoparticles to capture and store inhaled H_2_ gas as solid PdH, which then catalytically hydrogenate cytotoxic hydroxyl radicals (•OH) into H_2_O (Fig. 3) [78]. Instead of carrying H_2_, the Pd design rapidly captured liver-passing H_2_ during inhalation of 4 % H_2_ gas, mitigating H_2_'s inherent high dispersion and low solubility (1.6 mg/L), which would otherwise have required prolonged, unfeasible inhalation of concentrated H_2_. The Pd-based design also gave rise to sustained catalytic antioxidant activity through formation of PdH, whereas the free H_2_ would have been unlikely to facilitate. In a NASH mouse model, intravenous Pd nanoparticles followed by H_2_ gas inhalation significantly improved outcomes, enhancing both prevention and treatment models (mild or moderate NASH). As a result, lipid metabolism was improved through reduction of total liver cholesterol and triglycerides, liver enzymes alanine aminotransferase and aspartate aminotransferase decreased, collagen deposition reduced, and serum inflammatory cytokines reduced. The nanoplatform also displayed optimistic excretion after performing its therapeutic roles, where GSH injection further accelerated its clearance (liver Pd content reduced to ∼3% after 12 weeks).

Additionally, studies indicated that molecular hydrogen improve NAFLD by reducing oxidative stress and increasing hepatic PPARα and PPARγ [79]. Furthermore, hydrogen-rich water alleviated key features of liver disease, including cell death, inflammation, and fibrosis through a mechanism independent of the HO-1/IL-10 pathway [80]. These findings highlighted the complex mechanisms of hydrogen-based therapies, emphasizing their potential for new treatment approaches in treating liver diseases.

### 2.3 Therapeutic potential of NO in liver fibrosis

**Fig. 4:**
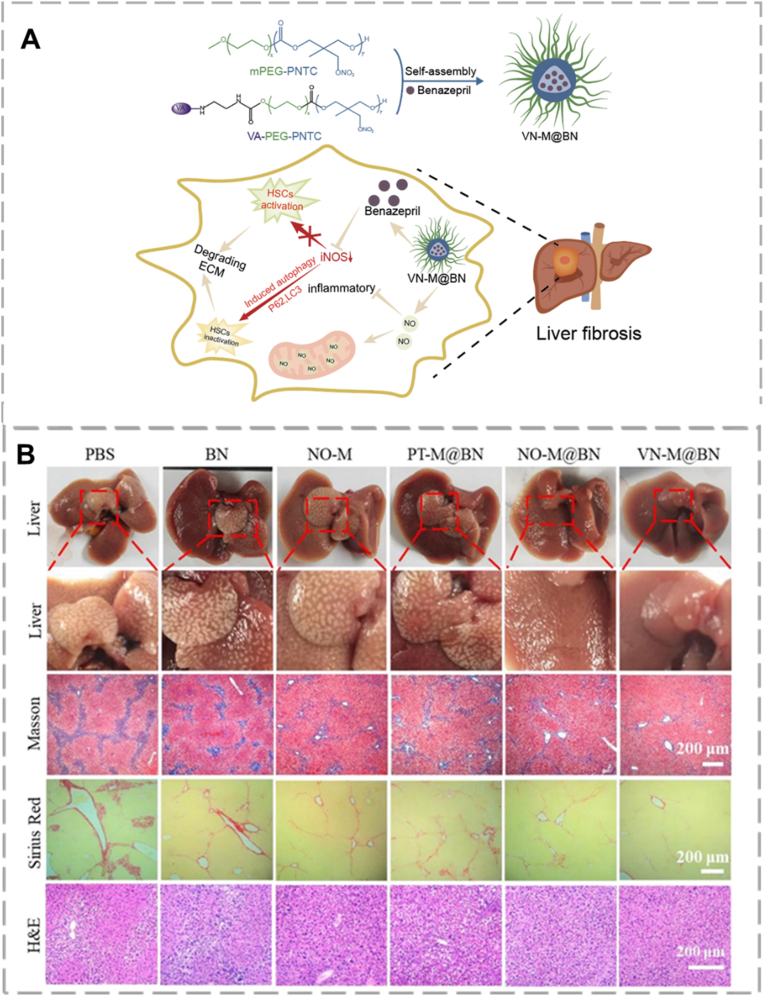
(A) Schematic illustration of NO's anti-fibrotic role in liver fibrosis. (B) Photos of liver tissue and histology sections stained with Masson's trichrome, Sirius Red, and H&E, from Ref. [86] with permission. (For interpretation of the references to color in this figure legend, the reader is referred to the Web version of this article.)

NO is an essential gaseous signaling molecule involved in physiological regulation. Its deficiency is a recognized factor in the development of various disorders, including diabetes, liver fibrosis, cardiovascular disease, neurodegeneration, and certain cancers [81]**.** It is produced endogenously, directly inhibiting HSC activation and reducing their pro-survival autophagic responses [82]. In healthy hepatocytes, inducible nitric oxide synthase (iNOS) catalyzes NO production from L-arginine. This NO then acts through a paracrine mechanism to decrease endothelial-induced HSC contraction, representing a key natural anti-fibrotic pathway [83,84]. Because HSC activation is a hallmark of liver fibrosis, NO's strong pro-apoptotic effect on HSCs highlights its potential as an anti-fibrotic agent. Liang and colleagues developed nanoparticles that specifically target HSCs and release NO locally when exposed to near-infrared (NIR) light, showing promise for treating liver fibrosis [85]. Similarly, in 2025, Yang and colleagues created a vitamin-A modified benazepril (BN)-loaded micelle (VN-M@BN) through the self-assembly of a poly(ethylene glycol)-*block*-poly(nitrate carbonate) copolymer [86]. The vitamin A ligand provided targeting towards retinol-binding protein receptor on HSCs, while the overall polymeric platform allowed targeting of fibrotic liver tissue through GSH-triggered response via its poly(nitrate carbonate) structure. The micelle nanocarrier further allowed for controlled sustained release of NO over 48 h under reducing (GSH) conditions, whereas free NO donors (nitrates) release the gas load prematurely within minutes. At target sites, BN worked to block the renin-angiotensin-aldosterone system, while the released NO directly downregulated α-smooth muscle actin (α-SMA), inducing apoptosis of activated HSCs. The results indicated that VN-M@BN was stable under circulation and effectively targeted fibrotic liver tissues. This targeted approach significantly reduced collagen buildup and slowed fibrosis in the injured liver (Fig. 4). By enabling precise drug delivery to activated HSCs, the NO-based nano-therapy enhanced anti-fibrotic effects while minimizing potential side effects.

### 2.4 Therapeutic potential of H_2_S in liver fibrosis

**Fig. 5:**
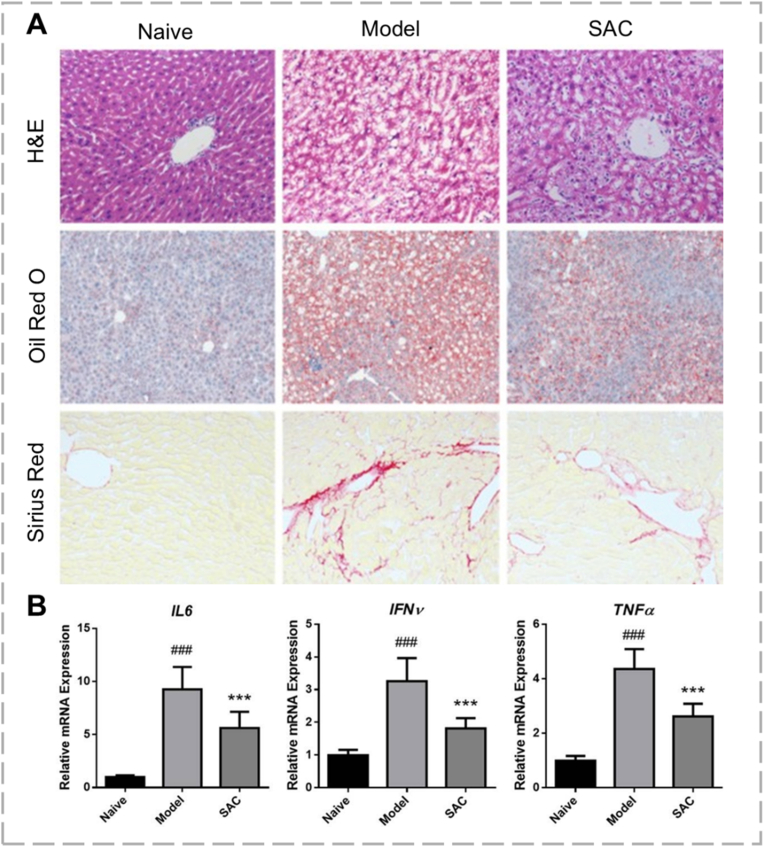
(A) SAC treatment attenuated CCl_4_-induced liver fibrosis, as demonstrated by representative histopathological evaluation using H&E, Sirius Red, and Oil Red O staining. (B) SAC modulated the expression of key genes related to inflammation, including interleukin-6 (IL-6), interferon-γ (IFN-γ), and tumor necrosis factor-α (TNF-α), reproduced from Ref. [90] with permission. (For interpretation of the references to color in this figure legend, the reader is referred to the Web version of this article.)

H_2_S is an endogenous gaseous mediator whose dysregulated metabolism contributes to fibrotic disease development. Studies demonstrated that supplementation with exogenous H_2_S donors has anti-fibrotic effects. These effects occur through H_2_S's interaction with key signaling pathways and ion channels, resulting in the inhibition of pro-fibrotic processes [87]. The importance of H_2_S in liver disease is presently well recognized [88]. However, the exact cellular and molecular mechanisms of H_2_S action are still not fully understood. Because of its vital role in various biological functions, H_2_S is broadly acknowledged as a fundamental gaseous signaling molecule [37]. S-allyl-cysteine (SAC), a primary component of aged garlic extract derived from S-alk(en)yl-cysteine sulfoxides, is a confirmed endogenous H_2_S donor [89]. In their study of its therapeutic potential, Gong et al. showed that SAC (50 mg/kg/day) significantly reduce CCl_4_-induced liver fibrosis in a rat model [90]. The treatment improved histopathological indicators, evidenced by lower fibrosis scores (H&E, Oil Red O, Sirius Red), as shown in Fig. 5. Mechanistically, SAC decreased hepatic mRNA levels of key inflammatory and fibrogenic cytokines, including IL-6, IFN-γ, TNF-α, and TGF-β, while increasing antioxidant enzymes such as superoxide dismutase, catalase, and glutathione peroxidase. A growing body of research suggests that insufficient endogenous H_2_S production promotes fibrosis in humans, while H_2_S supplementation offers protection against fibroproliferative diseases across multiple organs through anti-inflammatory, antioxidant, and anti-fibrotic mechanisms [91,92].

### 2.5 Therapeutic potential of SO_2_ in liver fibrosis

**Fig. 6:**
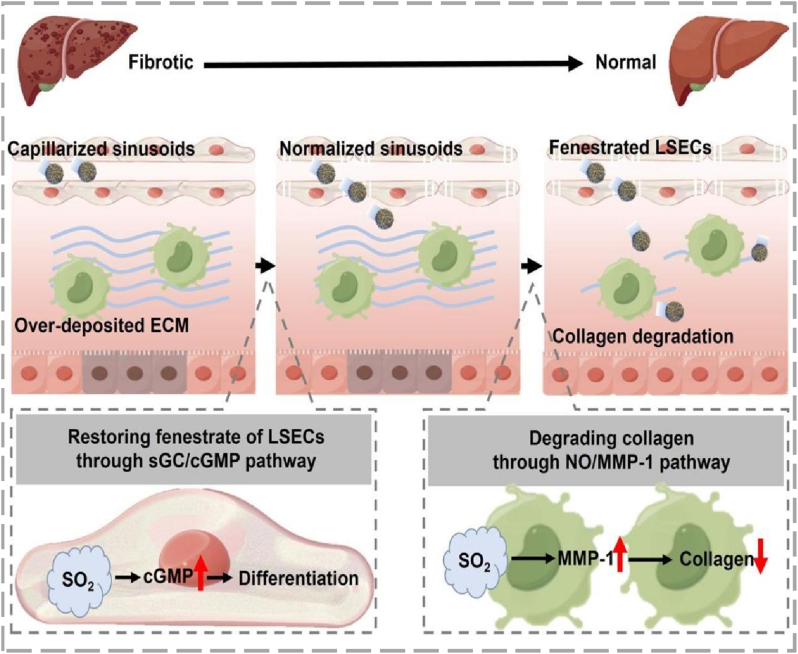
Schematic illustration of SO_2_-Releasing nanomotor synthesis and anti-fibrotic treatment strategy, reproduced from Ref. [96] with permission.

SO_2_ is recognized as an endogenously produced gaseous signaling molecule with significant therapeutic potential [93]. In the context of fibrosis, endogenous SO_2_ has been shown to inhibit the progression of fibrotic remodeling in organs such as the liver and lungs by reducing key processes like HSC activation and collagen deposition. Its protective mechanisms, which include vasodilation, suppression of the TGF-β1/Smad pathway, and reduction of oxidative stress and inflammation, make it a promising candidate for anti-fibrotic therapy [94,95]. To improve the effectiveness of SO_2_ gas-based therapy, Chen et al. designed for SO_2_-releasing nanomotors that respond to liver damage (Fig. 6). These nanomotors possessed an L-cysteine derivative, which acted as substrate to respond to the fibrotic liver's unique enzymatic concentration gradient involving cysteine dioxygenase and aspartate aminotransferase, thereby enhancing the retention of therapeutics at the disease sites via chemotaxis. Through enzymatic catalysis at fibrotic regions, SO_2_ was released, which further promoted both retention of the nanomotors and treatment of liver damage. Mechanistically, the released SO_2_ operated through two main pathways: it increased cyclic guanosine monophosphate (cGMP) to restore the normal morphology of liver sinusoidal endothelial cells (LSECs), and it stimulated NO production to activate matrix metalloproteinase-1 (MMP-1) for collagen breakdown and overcoming the excessive ECM barrier that hindered therapeutic penetration. Supporting this dual mechanism, in vivo experiments confirmed effective nanomotor retention and a significant reversal of liver fibrosis, with reduction of fibrotic lesions from 5.1% to 0.9% [96].

Currently, research on the relationship between endogenous SO_2_ and fibrosis is limited and mostly regarding myocardial fibrosis. Building on this, understanding the physiological roles and mechanisms of endogenous SO_2_ in fibrotic processes would therefore open up important therapeutic implications beyond the heart. Further studies may reveal its potential to reduce fibrosis in various organ systems [64].

### 2.6 Therapeutic potential of O_2_ in liver fibrosis

**Fig. 7:**
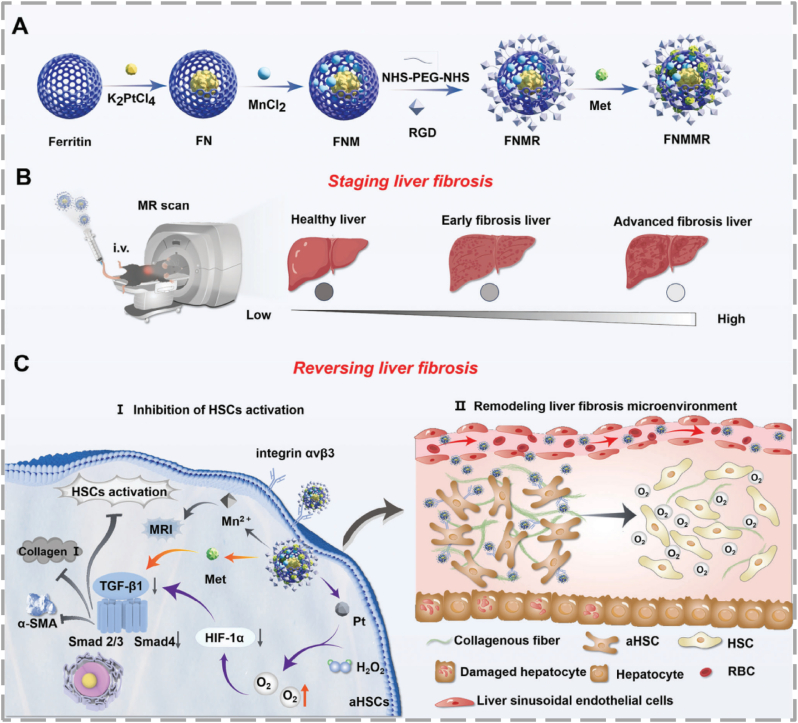
(**A)** Schematic illustrating the fabrication process of FNMMR. (**B)** The mechanism by which FNMMR enables concurrent staging and visualization of liver fibrosis through non-invasive MRI. (**C)** Depiction of the multi-targeted therapeutic action of FNMMR against fibrotic tissue, reproduced from Ref. [99] with permission.

Molecular O_2_ is also being utilized through innovative methods. By using targeted delivery, scientists are tackling hepatic hypoxia, a low O_2_ condition that activates scar-producing HSCs. This offers an innovative approach to protecting the liver from initial injuries that lead to fibrosis. Ferritin, an endogenous iron storage protein, has become a key drug delivery vehicle because of its unique spherical nanocage structure and natural biocompatibility [97,98]. Cui et al. engineered a theranostic nanoplatform, RGD-functionalized and metformin-loaded ferritin-platinum-manganese, for MRI and targeted therapy of liver fibrosis [99]. The nanocage naturally showed catalase-like activity, breaking down endogenous hydrogen peroxide (H_2_O_2_) into O_2_ to improve the hypoxic tumor microenvironment (Fig. 7). This oxygen generation reduced HIF-1α, which in turn suppressed the pro-fibrotic TGF-β1/Smad signaling pathway. This effect was strengthened by the co-encapsulated metformin, which worked synergistically to inhibit the same pathway, leading to effective suppression of HSC activation and a notable reduction in collagen build-up. Importantly, extensive in vivo safety assessments, including tissue and blood analyses, confirmed the high biocompatibility and low off-target toxicity of this nanoplatform.

### 2.7 Other gases with potential in liver fibrosis

The therapeutic potential of gases in liver fibrosis is expanding beyond the traditional players like CO, NO, H_2,_ H_2_S, SO_2_, and O_2_. Among other relatively less investigated gases, methane (CH_4_) has shown anti-inflammatory properties in experimental models. Its administration may be able to slow the progression of fibrosis under certain conditions [100,101]. Despite the relatively little direct work on liver fibrosis, methane had been examined for liver injuries. Methane-rich saline was found to reduce liver inflammation and fibrosis in mice, through downregulating the TLR4/NF-*κ*B signaling pathway and NLRP3 protein activation [102]. For liver injury under sepsis, methane-rich saline similarly reduced inflammatory responses and reduced oxidative damage in mice through TLR4/NF-*κ*B signaling [103]. For carbon tetrachloride (CCl_4_)-induced liver injury, methane also exhibited anti-inflammatory effects [104], likely converging at NF-κB signaling as well, revealing a growing consensus on how methane acts at the injured liver, regardless of initial cause of the damage [105]. In a case of pulmonary fibrosis, a 2026 paper reported the use of methane prodrug Fe(BPY)_2_(CH_3_)_2_ assembled with PLGA-PEG copolymer to control methane gas release at the lungs, reducing inflammation and fibrosis progression [106]. Similar to methane, ammonia (NH_3_) also holds complex roles in liver fibrosis, but it is far more recognized as a toxic consequence of disease and instead be exploited for targeting [107] or for scavenging instead [108]. In a mice model of NAFLD, ornithine phenylacetate was used to scavenge ammonia with resultant significant prevention of fibrotic progression [109]. Therefore, in addition to delivering gases, the scavenging of gases or inhibition of production of gases can also be relevant when relating gases to advanced delivery methods.

## 3 Discussion and future perspectives

Medical gas therapy could fundamentally revolutionize the treatment of liver fibrosis. Gases, such as CO, H_2_, NO, H_2_S, and SO_2_, possess the unique and inherent endogenous property of being able to affect multiple elements of the disease pathological process [64]. Unlike most drugs that target a single pathway, these gasotransmitters can simultaneously reduce inflammation, combat oxidative damage, and directly deactivate the HSCs responsible for scar tissue production [110]. This multi-pronged approach can be vital for breaking the vicious cycle of scarring that propels fibrosis forward. A major breakthrough has been the creation of advanced delivery systems, such as targeted nanoparticles and even self-propelled nanomotors [111]. These nanoscale carriers address the longstanding problems associated with gas therapy, like gases being too short-lived, too toxic due to off-target effects, or simply failing to reach the right cells. For example, CO-releasing micelles that alleviate low oxygen conditions in the liver, and SO_2_-releasing nanomotors that assist in repairing damaged blood vessels have been designed [112,113]. These developments support the advance of medical gases toward real, targeted treatments.

Thereafter, the path to the clinic should involve overcoming several key challenges. The next step may be to develop even smarter, stimuli-responsive delivery systems that can detect the disease environment and release their gas cargo only when and where it is needed, such as in response to high levels of certain enzymes or oxidative stress in the damaged liver. Accordingly, single stimuli may prove insufficient or too primitive for targeted delivery to fibrotic lesions. Elevated ROS is not unique to fibrotic lesions and may lead to off-target release at other less important inflamed areas within the body, building undesirable side effects over time. Multifunctional designs can consider response to two or more stimuli before release of gaseous load (high H_2_O_2_ due to inflamed hepatocytes and also high TGF-β activity under activated HSCs). Ongoing research also needs to focus on identifying optimal doses of each gas, if possible, and what off-target effects will encompass, due to the known multifunctional endogenous roles of the gases throughout the body. The design of nanomedical systems for releasing/generating gases is relatively new and unexplored with regards to clinical translation and therefore, biosafety-related studies focusing on biodistribution, long-term fate, and stability of the gas release, may be particularly valuable. Further extensions of developing medical gases for liver fibrosis will likely involve comparisons against clinical drugs, such as Resmetirom and SGLT2/GLP-1 agonists, in terms of fibrosis staging and clinical standards of assessment. By extension, the potential to combine different gases in a single treatment will also be valuable to explore for a synergistic therapeutic effect. A single nanoparticle carrying two gas donors could target fibrosis through multiple pathways simultaneously, potentially yielding better results with lower, safer doses. Turning this potential into reality will require a collaborative effort that combines expertise in material science, safety testing, and ultimately, rigorous clinical trials in patients.

## 4 Conclusion

In conclusion, combining medical gas therapy with advanced nanotechnology provides a new and powerful approach to combat liver fibrosis. By exploiting the body's natural gaseous signaling molecules and employing precise delivery methods to target the disease site, liver fibrosis treatment may move beyond symptom management to actively reversing scar tissue. Although the journey from research to patient treatment is still in progress, the promising results from animal studies and the rapid pace of innovation provided strong reasons to remain hopeful for a new class of effective, targeted therapies for this common disease.

## CRediT authorship contribution statement

**Peiyuan Tian:** Data curation, Writing – original draft, Writing – review & editing. **Jinping Wang:** Data curation, Visualization, Writing – review & editing. **Haohan Yang:** Data curation, Visualization. **Zhenpu Jiang:** Data curation, Investigation. **Jinshuai Xu:** Data curation, Investigation. **Xueli Xu:** Conceptualization, Data curation, Investigation. **Nengyi Ni:** Data curation, Formal analysis, Writing – review & editing. **Teng Liu:** Conceptualization, Supervision, Writing – review & editing. **Xiao Sun:** Conceptualization, Funding acquisition, Project administration, Resources, Supervision.

## Declaration of competing interest

The authors declare that they have no known competing financial interests or personal relationships that could have appeared to influence the work reported in this paper.

## Data Availability

No data was used for the research described in the article.
